# Emerging MDR-*Pseudomonas aeruginosa* in fish commonly harbor *opr*L and *tox*A virulence genes and *bla*_TEM_, *bla*_CTX-M_, and *tet*A antibiotic-resistance genes

**DOI:** 10.1038/s41598-020-72264-4

**Published:** 2020-09-29

**Authors:** Abdelazeem M. Algammal, Mahmoud Mabrok, Elayaraja Sivaramasamy, Fatma M. Youssef, Mona H. Atwa, Ali W. El-kholy, Helal F. Hetta, Wael N. Hozzein

**Affiliations:** 1 Bacteriology, Mycology and Immunology Department, Faculty of Veterinary Medicine, Suez Canal University, Ismailia, 41522 Egypt; 2 Department of Fish Diseases and Management, Faculty of Veterinary Medicine, Suez Canal University, Ismailia, 41522 Egypt; 3 Present Address: Department of Veterinary Microbiology, Faculty of Veterinary Science, Chulalongkorn University, Bangkok, 10330 Thailand; 4 Department of Clinical Pathology, Ismailia Branch, Animal Health Research Institute, Ismailia, 41522 Egypt; 5 Department of Medical Microbiology and Immunology, Faculty of Medicine, Assuit University, Assuit, 71515 Egypt; 6 Department of Internal Medicine, College of Medicine, University of Cincinnati, Cincinnati, OH 45267-0595 USA; 7 Zoology Department, College of Science, King Saud University, Riyadh, 11451 Saudi Arabia; 8 Botany and Microbiology Department, Faculty of Science, Beni-Suef University, Beni-Suef, 62511 Egypt

**Keywords:** Microbiology, Molecular biology, Diseases

## Abstract

This study aimed to investigate the prevalence, antibiogram of *Pseudomonas* *aeruginosa* (*P.* *aeruginosa*), and the distribution of virulence genes (*opr*L*,* *exo*S, *phz*M, and *tox*A) and the antibiotic-resistance genes (*bla*_*T*EM_, *tet*A, and *bla*_CTX-M_). A total of 285 fish (165 *Oreochromis* *niloticus* and 120 *Clarias* *gariepinus*) were collected randomly from private fish farms in Ismailia Governorate, Egypt. The collected specimens were examined bacteriologically. *P*. *aeruginosa* was isolated from 90 examined fish (31.57%), and the liver was the most prominent infected organ. The antibiogram of the isolated strains was determined using a disc diffusion method, where the tested strains exhibited multi-drug resistance (MDR) to amoxicillin, cefotaxime, tetracycline, and gentamicin. The PCR results revealed that all the examined strains harbored (*opr*L and *tox*A) virulence genes, while only 22.2% were positive for the *phz*M gene. On the contrary, none of the tested strains were positive for the *exo*S gene. Concerning the distribution of the antibiotic resistance genes, the examined strains harbored *bla*_TEM_, *bla*_CTX-M_*,* and *tet*A genes with a total prevalence of 83.3%, 77.7%, and 75.6%, respectively. Experimentally infected fish with *P.* *aeruginosa* displayed high mortalities in direct proportion to the encoded virulence genes and showed similar signs of septicemia found in the naturally infected one. In conclusion, *P.* *aeruginosa* is a major pathogen of *O.* *niloticus* and *C.* *gariepinus.* *opr*L and *tox*A genes are the most predominant virulence genes associated with *P.* *aeruginosa* infection. The *bla*_CTX*-M*_*,* *bla*_TEM_, and *tet*A genes are the main antibiotic-resistance genes that induce resistance patterns to cefotaxime, amoxicillin, and tetracycline, highlighting MDR *P.* *aeruginosa* strains of potential public health concern.

## Introduction

*Pseudomonads* are one of the most threatening fish pathogens that induce ulcerative syndrome and hemorrhagic septicemia^1^. Various bacterial pathogens affect a wide range of aquatic species and are responsible for considerable economic losses, worldwide. It was estimated that up to 50% of farms fish are lost due to bacterial infection before their marketing. The economic losses are mainly attributed to poor growth, high mortalities, and improper flesh quality^2–5^. *Pseudomonas* *aeruginosa* is a part of normal fish microbiota, however under stressful conditions such as malnutrition and overcrowding the bacteria have become highly opportunistic and pathogenic, causing serious illness including, hemorrhagic septicemia, gill necrosis, abdominal distension, splenomegaly, friable liver, and congested kidney^6^.

*P.* *aeruginosa* is an aerobic Gram-negative motile, non-spore-forming rods, catalase, and oxidase-positive^7^. Besides, this bacterium possesses many virulence-related determinants including cell-mediated and secreted virulence types. The cell-mediated types include; pilli, flagella, and lipopolysaccharide (LPS) which are commonly involved in bacterial colonization and motility, delivery of active proteins into the host cells, and establishment of persistent infections^8^. Likewise, the secreted virulence factors fortify the inflammatory processes, induce severe tissue damages, facilitate bacterial invasion and dissemination, and accelerate the progression of diseases^9^.

Indeed, the identification of *Pseudomonas* spp. is essential for accurate diagnosis, outbreaks prediction, and preventive and/or prophylactic measures implementation in aquaculture^10^. Like most of invading pathogens, phenotypic characterization of *P.* *aeruginosa* required protracted and complicated steps of morphological and biochemical identifications, which were intractable, time-wasting, and occasionally unreliable^11^. Therefore, the molecular-based detection of this pathogen could help to obtain a full idea about the ecological importance of such pathogens and rescind the shortcomings of conventional methods^12^. Most of the *Pseudomonas* species showed different resistance patterns against various antimicrobial agents^13^. Specifically, *P.* *aeruginosa* could develop innate and/or acquired resistance to several antimicrobial agents due to the active efflux of antibiotics and the permeability of its outer membrane^9^. Thus, the molecular typing of most inherited antibiotic-resistance genes should be performed to avoid the emergence of antibiotic-resistant strains that have a global health concern^14^.

Nowadays, serious attempts for further detection of *P.* *aeruginosa* were considered not only for its economic impact but also for its public health importance. *Pseudomonas* infection, generally induced by *P.* *aeruginosa* has public health concern and causes healthcare-associated illnesses for many consumers^15^. *Pseudomonas* species are presently cataloged as a food-borne illness that affects humans by consuming spoiled foods and ready-to-eat products, as well as manipulating the contaminated seafood^16,17^. Although few studies have claimed possible routes of disease transmission, people with weakened immune systems or those works with infected fish are usually at risk^6^. *P.* *aeruginosa* infection could occur in humans via the consumption of raw fish and its byproducts^18^. It is reported that the typical colonies of *P.* *aeruginosa* isolated from fish are closely related to those causing hospital-acquired pneumonia in humans^17^.

The application of the antimicrobial susceptibility testing is ultimately required for disease control. Globally, the extensive use of antimicrobial agents in the aquaculture sector, as well as transmission of multidrug-resistant bacterial pathogens between terrestrial and aquatic ecosystems have been reported^19^. The antibiotic resistance mechanism mostly occurs due to the transfer of R-Plasmid^20^. Various reports investigated the occurrence and transmission of antibiotic resistance between human and animal food^21^, with less attention to fish, so that fewer data are available about the antimicrobial resistance in fish^22^.

Several virulence factors are commonly associated with *P.* *aeruginosa* especially exotoxin A and exotoxin S that are controlled by a cell to cell signaling patterns, in addition, exotoxin A is responsible for the inhibition of protein-biosynthesis, while Exotoxin S is a bi-functional protein that enhances the ribosyltransferase and GTPase activity resulting in cell-apoptosis^23^. Furthermore*,* *P.* *aeruginosa* produces phenazine compounds, which are biologically active substances involved in bacterial competitiveness and virulence in both human and animal hosts^24^. The outer membrane proteins (L-lipoproteins) of *P.* *aeruginosa* are incriminated in the bacterial resistance to antiseptics and antibiotics^25^. The resistance of *P.* *aeruginosa* to the β-lactam antibiotics including penicillin (1st, 2nd, and 3rd generations) and cephalosporin (such as cefotaxime) is mainly attributed to the Extended Spectrum Beta-lactamases (ESBLs). *bla*_CTX-M_ and *bla*_TEM_ are the main Extended Spectrum β-lactamases genes that induce such type of resistance^26^.

In this context, the present study aimed to identify the phenotypic characteristics of *P.* *aeruginosa* strains, retrieved from two freshwater cultured fish species (*Oreochromis* *niloticus* and *Clarias* *gariepinus*) and their antibiogram. Furthermore, the genotypic characterization of the virulence genes: *oprL;* outer membrane lipoprotein-L*,* *exo*S; exotoxin S gene, *phz*M; phenazine-modifying gene, and *tox*A; exotoxin A gene as well as the antibiotic-resistance genes: *bla*_TEM_ and *bla*_CTX-M_; ESBLs genes, and *tet*A; tetracycline resistance gene was performed to examine their potential for bacterial pathogenicity and probe their potential mechanisms of resistance to commercially available antibiotics.

## Methods

### Animal ethics

All methods were carried out following relevant guidelines and regulations. Handling of fish and all the experimental protocols carried out by well-trained scientists according to the guidelines of the Animal Ethics Review Committee of Suez Canal University. All used protocols are approved by Suez Canal University, Egypt.

### Fish sampling

A total of 285 fish samples represented as 165 *O.* *niloticus* (70 apparently healthy and 95 moribunds) and 120 *C.* *gariepinus* (55 apparently healthy and 65 moribunds) were collected randomly; (5 sampling points at two months interval) from two private freshwater farms located at Ismailia Governorate, Egypt over the period of July 2018 to February 2019. The farms implemented semi-intensive culture system and applied the same management and treatment programs. Based on the clinical history, the farms routinely used different antibacterial agents such as amoxicillin, cefotaxime, tetracycline, norfloxacin, and gentamicin for regular prophylactic and treatment protocols. Fresh specimens of apparently healthy and moribund fish were transferred alive in aerated plastic bags to the laboratory of Microbiology, Faculty of Veterinary Medicine, Suez Canal University for further clinical and bacteriological examinations. Samples from liver, kidneys, and spleen were collected aseptically following the protocols of Yanong^27^.

### Clinical and postmortem inspections

Fish were clinically examined for the presence of external and internal lesions according to Austin et al.^28^. Briefly, the fish were examined in a sterile manner using a three-line incision in the case of *O.* *niloticus* or a V-shaped incision in the case of *C.* *gariepinus*. Antiseptic treatment of the skin was performed using 70% ethyl alcohol. The incision was carried out with sharp pointed scissors, which were introduced into the anus in such manner that the intra-abdominal point remained steady while the cut was made in close contact with the ventral side to avoid internal tissue damage. The abdominal wall was removed; the internal organs were exposed and examined macroscopically for any gross abnormalities.

### Isolation and identification of *P*. *aeruginosa*

A loopful sample from the collected internal organs was directly streaked onto cetrimide agar and MacConkey's agar (Oxoid, UK), and left incubated at 37 °C for 24 h under aerobic condition. The production of yellowish-green fluorescent pigment is commonly associated with *Pseudomonads*^29^. All suspected colonies were harvested and purified for phenotypic and biochemical characteristics. Briefly, all isolates were identified morphologically using Gram's stain and biochemically using various biochemical tests; catalase, oxidase, urease, indole, methyl red, Voges Proskauer, citrate utilization, H_2_S production, mannitol fermentation, and gelatin hydrolysis, as well as for their motility using hanging drop technique according to Mac Faddin^30^. Furthermore, the identified isolates were confirmed using a species-specific set of primers (PaF: 5′-GGGGGA TCTTCGGACCTCA-3′; PaSR: 5′-TCCTTAGAGTGCCCACCCG-3′) targeting 16S rRNA gene of *P.* *aeruginosa* as described elsewhere by Spilker et al.^31^.

### Antimicrobial susceptibility testing

The susceptibility of the retrieved isolates to different commercial antimicrobial agents (Oxoid), including ofloxacin (5 µg), amoxicillin (10 µg), cefotaxime (30 µg), tetracycline (30 µg), levofloxacin (5 µg), gentamicin (10 µg), norfloxacin (10 µg), tobramycin (10 µg), and colistin sulfate (25 µg) was evaluated using a disc diffusion method^32^. The selected antimicrobial agents are representatives of the drugs used commonly in the aquaculture sector in Egypt and were selected according to the National Antimicrobial Resistance Monitoring System records. The test was performed using Muller Hinton agar plates (Oxoid, UK) and the plates were incubated at 37 °C for 24 h. The test was conducted according to the instructions of the Clinical Laboratory Standards Institute (CLSI) criteria^33^.

### Molecular typing of the virulence and antibiotic-resistant genes of the isolated *P. aeruginosa* strains

DNA of purified strains was extracted using a silica-based membrane QIAamp DNA Mini kit (Catalogue no. 51304) according to the manufacturer’s instructions. Genomic DNA templates were quantified using Nanodrop (Nanodrop 1000, Thermo Scientific, UK), adjusted to 100 ng μL^−1^, and stored at − 20 °C until used for PCR. Ninety representative *P.* *aeruginosa* strains (the same strains that were tested for the antimicrobial susceptibility) were tested for the detection of virulence genes, four sets of primers targeting (*opr*L*,* *exo*S, *phz*M, and *tox*A) genes were selected based on the previous publications of Xu et al.^34^, Winstanley et al.^35^, Finnan et al.^36^, and Matar et al.^37^, respectively. Further, to verify the resistance of retrieved strains to the commercially available antibiotics, three sets of primers targeting *bla*_TEM_, *tet*A, and *bla*_CTX-M_ genes were also selected according to Colom et al.^38^, Randall et al.^39^, and Fazeli et al*.*^40^, respectively. All primers supplied by (Metabion Company, Germany), and their oligonucleotides sequences and PCR conditions are given in Table 1. PCR reactions (25 μL) were amplified in T100 Gradient Thermocycler (Biometra, Jena, Germany) using EmeraldAmp GT PCR Master Mix (Code No. RR310A, Takara, Japan). A reaction with no template DNA was used as a negative control, while a virulent reference strain of *P.* *aeruginosa* (multidrug-resistant to amoxicillin, cefotaxime, tetracycline, and gentamicin), kindly provided by Animal Health Research Institute in Dokki, Cairo, Egypt, was used as positive control. The products were screened by horizontal 1.5% (w/v) agarose gel electrophoresis (AppliChem GmbH, Darmstadt, Germany) and then photographed.

**Table 1:** List of oligonucleotide sequences and their PCR conditions used in the current study.

Target gene	Primer sequences 5′-3'	Amplicon (bp)	PCR conditions					References
			No of cycles	Denaturation	Annealing	Extension	Final Extension	
*opr*L	ATG GAA ATG CTG AAA TTC GGC CTT CTT CAG CTC GAC GCG ACG	504	40	96 ˚C for 1 min	55 ˚C for 1 min	72 ˚C for 1 min	72˚C for 10 min	^34^
*tox*A	GACAACGCCCTCAGCATCACCAGC CGCTGGCCCATTCGCTCCAGCGCT	396	30	94 ˚C for 1 min	55 ˚C for 1 min	72 ˚C for 1 min		^37^
*exo*S	GCGAGGTCAGCAGAGTATCG TTCGGCGTCACTGTGGATGC	118	36	94 ˚C for 30 s	58 ˚C for 30 s	68 ˚C for 1 min		^35^
*phz*M	ATGGAGAGCGGGATCGACAG ATGCGGGTTTCCATCGGCAG	875	30	94 °C for 30 s	54 ˚C for 30 s	72 ˚C for 1 min		^36^
*bla*_TEM_	ATCAGCAATAAACCAGC CCCCGAAGAACGTTTTC	516	32	94 °C for 30 s	54 ˚C for 30 s	72 ˚C for 1 min		^38^
*tet*A	GGTTCACTCGAACGACGTCA CTGTCCGACAAGTTGCATGA	576	30	94 ˚C for 1 min	56 ˚C for 30 s	72 ˚C for 1 min		^39^
*bla*_CTX-M_	ATGTGCAGYACCAGTAARGT TGGGTRAARTARGTSACCAGA	593	35	95 ˚C for 45 s	51 ˚C for 45 s	72 ˚C for 1 min		^40^

### Pathogenicity test

#### Acclimation period

A total of 150 healthy *O.* *niloticus* weighing 45 ± 3 g with no history of previous infections were collected randomly from Fish Research Center, Suez Canal University, Egypt, and left acclimated in a clean fiberglass tank of 1 m^3^ holding capacity for 15 days prior to the challenge. *Oreochromis* *niloticus* was selected as a model for the present study due to its local availability and ease of cultivation, handling, and transportation. The tank was filled with sand-filtered, UV-sterilized, dechlorinated tap water with an average salinity of 0.3 ± 0.1 g L^−1^. Dissolved oxygen was monitored at 5 ± 1 mg L^−1^ using automatic air suppliers (RINA, Genova, Italy), while the water temperature was maintained at 27 ± 0.52 °C. Tank pH was regulated at 7.5 and 13 h light/11 h dark cycle was adopted. Ammonia and nitrite were measured twice a week and never exceeded 0.05 and 0.25 mg L^−1^, respectively. The fish were fed two times daily (09:00 and 20:00 h) until visual satiety on a commercial pellet of 30% crude protein (Skretting, Alexandria, Egypt). The organic wastes and other debris were siphoned and 30% of the water was replaced daily to reduce the toxicity of ammonia. Fish that showed normal reflexes with no apparent lesions selected for the challenge trial.

#### Challenge trial

It was performed according to the directive on the protection of animals used for scientific purposes and following the ethical approval sheet mentioned above. One hundred and twenty of acclimated *O.* *niloticus* were equally distributed into three groups; each contributed 2 glass aquaria of 80 L and 20 fish holding capacity. The trial was performed in duplicates (*n* = 2). The fish of the first group (C) were injected intraperitoneally (IP) with 100 µL of sterile phosphate buffer saline and served as a control, while the fish of the other groups (T1 and T2) were injected with 100 µL of the overnight culture of virulent *P.* *aeruginosa* strains (A and B, respectively) at a concentration of 3 × 10^7^ cells mL^−1^ ^1^. The isolates were selected based on the fact that all recovered isolates have identical biochemical and molecular properties. One of these isolates (A) harbored *opr*L, *tox*A, and *phz*M virulent genes, while the other (B) just encoded the *opr*L and *tox*A genes. For inocula preparation, bacteria were routinely cultured on tryptic soy broth (Oxoid) at 37 °C for 24 h. Thereafter, the growing bacteria were adjusted to the desired concentration using 0.5 McFarland standards and by the Helber counting chamber. The pathological lesions and cumulative deaths were recorded daily among experimental groups for 14 days post-challenge. Moribund and freshly dead fish were collected, examined immediately to verify the cause of death. Mortalities were considered only when the injected strain was recovered from the experimentally infected fish (Koch’s postulates).

### Statistical analysis

The Chi-square was carried out to analyze the data to test the null hypothesis of various treatments using the statistical analysis software (SAS, Software version 9.4, SAS Institute, Cary, NC). The significance level was (*P* < 0.05).

## Results

### Clinical and postmortem findings

**Figure 1:**
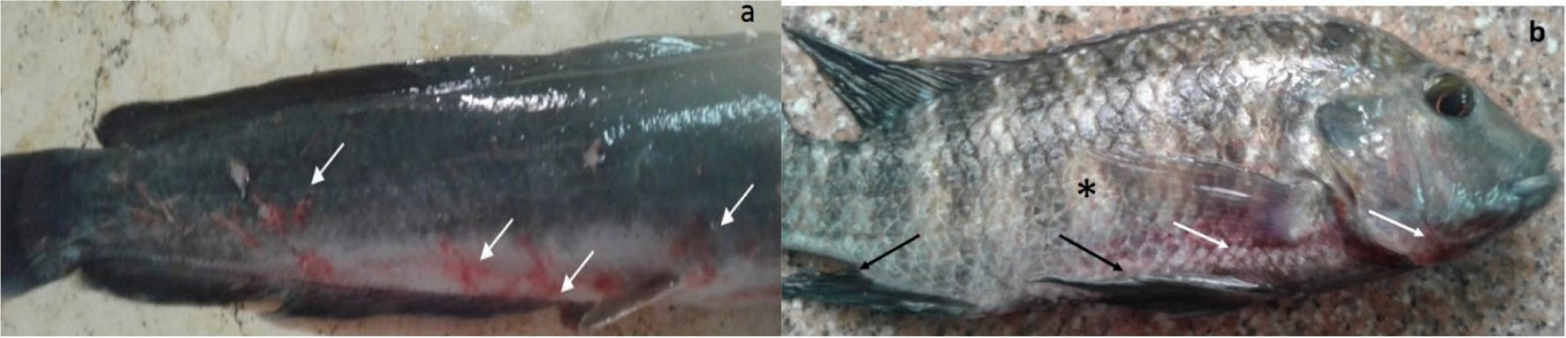
Clinical examination of naturally infected fish (**a**) catfish (*Clarias gariepinus*) showing irregular hemorrhages on external body surfaces, especially at the ventral part of the abdomen and around the vent (white arrows), (**b**) Nile tilapia (*Oreochromis niloticus*) showing scattered hemorrhagic spots (white arrows), detached scales (*), and fins erosion (black arrows).

**Figure 2:**
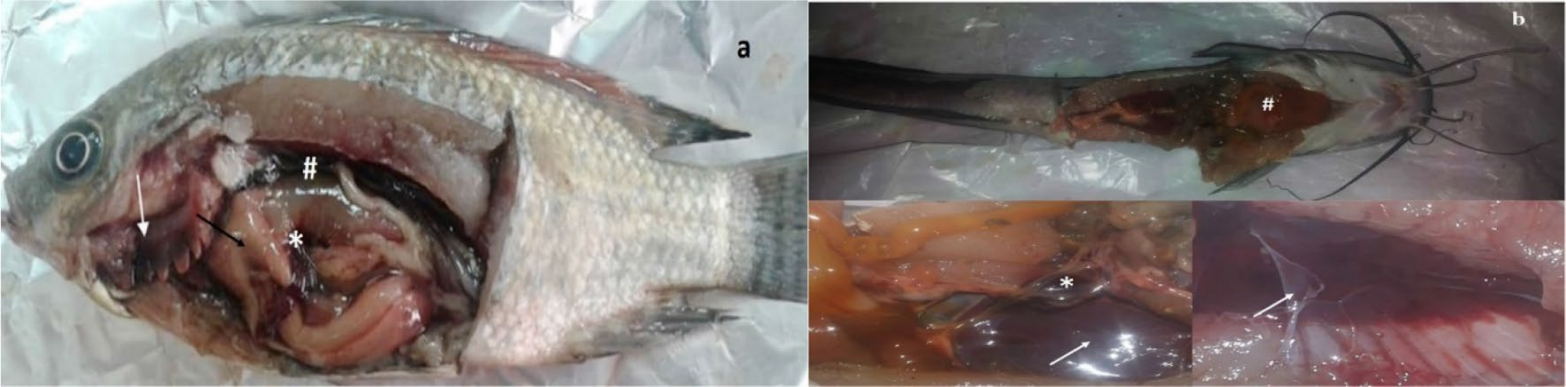
Post-mortem examination of naturally infected fish (**a**) Nile tilapia (*Oreochromis niloticus*) showing pale liver (black arrow), necrotic gills (white arrow), engorged spleen (*), serous fluid exudate, and congested kidney (#), (**b**) catfish (*Clarias gariepinus*) showing friable liver (#), congested kidneys (white arrows), and engorged spleen (*).

In the present study, a total of 285 fish samples represented as 165 *O.* *niloticus* and 120 *C.* *gariepinus* were collected randomly and examined. The clinical inspection revealed that 95 *O.* *niloticus* (95/165, 57.5%) and 65 *C.* *gariepinus* (65/120, 54.1%) were moribund and showed the signs of hemorrhagic septicemia. Regardless of the fish species, most of the naturally infected fish shared the same typical clinical signs, including hemorrhages on external body surfaces, mainly at the ventral aspect of the abdomen and around the vent (Fig. 1a). Others showed fins erosions, skin darkness, and detached scales (Fig. 1b). Internally, the infected fish showed typical signs of hemorrhagic septicemia represented by pale liver, necrotic gills, engorged spleen, and abdominal dropsy with reddish ascetic exudates (Fig. 2a,b).

### Bacteriological assay

The bacteriological examination revealed that all retrieved isolates were motile, Gram-negative bacilli, arranged in double or short chains. The colonies reacted positively to catalase, oxidase, nitrate reduction, gelatin hydrolysis, citrate utilization, and mannitol fermentation, while they were negative for H_2_S production, urease, Voges Proskauer, indole, and methyl red. Based on the morphological and biochemical characteristics, all isolates were identified as *P.* *aeruginosa*. The typical isolates of *P.* *aeruginosa* displayed large irregular colonies with a fruity odor and produced a yellowish-green fluorescent pigment on cetrimide agar (C.A) at 37 °C for 24 h. The bacteria grew on MacConkey's agar and showed flat, smooth, non-lactose fermenting colonies with regular edge and alligator skin like from the top view. Furthermore, all isolates were positive for the PCR amplification of species-specific 16S rRNA gene. The total prevalence of *P.* *aeruginosa* among the examined fish was 31.57%. The highest prevalence was recorded in *O.* *niloticus* (54/165, 32.72%) followed by *C.* *gariepinus* (36/120, 30%). None of the examined apparently healthy fish yielded *P.* *aeruginosa*, moreover, *P.* *aeruginosa* was isolated from 54 moribund *O.* *niloticus* (54/95, 56.8%) and 36 moribund C. *gariepinus* (36/65, 55.4%). The statistical analysis demonstrated that there was no significant difference in the prevalence of *P.* *aeruginosa* in *O.* *niloticus* and *C.* *gariepinus* (*P* > 0.05) (Table 2). Regarding the prevalence of *P.* *aeruginosa* in various infected organs, the liver was the most prominent infected organ, followed by the kidney and spleen (Table 3). The presence of *P.* *aeruginosa* in at least one organ of the fish was considered positive for the bacterium. The statistical analysis showed a significant difference in the prevalence of *P.* *aeruginosa* among the internal organs of the examined fish (*P* < 0.05).

**Table 2:** Total prevalence of *P. aeruginosa* among the examined *O. niloticus* and *C. gariepinus*.

Fish species	No of Examined fish	Positive for *P. aeruginosa*			
		No	%	Chi-square value	*P-*value
*O. niloticus*	165	54	32.72	0.2392	0.6248
*C. gariepinus*	120	36	30		
Total	285	90	31.57

**Table 3:** Prevalence of *P. aeruginosa* in different organs of naturally infected *O. niloticus* and *C. gariepinus*.

Fish species	No of isolates	Organs						Chi-square value	*P*-value
		Liver		Kidney		Spleen			
		No	%	No	%	No	%		
*O. niloticus*	112	54	48.2	40	35.7	18	16.1	26.4643	< .0001
*C. gariepinus*	84	36	42.8	28	33.3	20	23.9	6.0952	0.0475
Total	196	90	45.9	68	34.7	38	19.4		

### Antibiogram of the isolated *P. aeruginosa* strains

The antimicrobial susceptibility testing was conducted on 90 representative isolates of *P.* *aeruginosa* (one isolate/each infected fish; isolated from the liver). As shown in Table 4, the tested strains showed different resistance patterns to various antimicrobial agents (*P* < 0.0001). The examined strains were entirely sensitive to colistin sulfate (100%), highly sensitive to norfloxacin (88.89%), while exhibited remarkable resistance to amoxicillin (83.3%), cefotaxime (77.7%), tetracycline (75.6%), and gentamicin (67.6%). Additionally, out of them, 50 strains (50/90, 55.5%) showed multi-drug resistance to four antimicrobial agents; amoxicillin, cefotaxime, tetracycline, and gentamicin (Table 6).

**Table 4:** Antimicrobial resistance profiles of *P. aeruginosa* strains (*n* = *90*) retrieved from naturally infected *O. niloticus* and *C. gariepinus*.

Antimicrobial agents	Concentration (µg)	Sensitive		Intermediate		Resistant	
		No	%	No	%	No	%
Ofloxacin	5	63	70	19	21.11	8	8.89
Amoxicillin	10	0	0	15	16.7	75	83.3
Cefotaxime	30	5	5.6	15	16.7	70	77.7
Tetracycline	30	0	0	22	24.4	68	75.6
Levofloxacin	5	11	12.22	66	73.33	13	14.44
Gentamicin	10	0	0	29	32.2	61	67.6
Norfloxacin	10	80	88.89	6	6.67	4	4.44
Tobramycin	10	0	0	70	77.8	20	22.2
Colistin sulfate	25	90	100	0	0	0	0
Chi-square value		642.2441		276.3880		352.3856	
*P*-value		< .0001		< .0001		< .0001	

### Molecular typing of the virulence and antibiotic-resistance genes of the isolated *P. aeruginosa* strains

**Figure 3:**
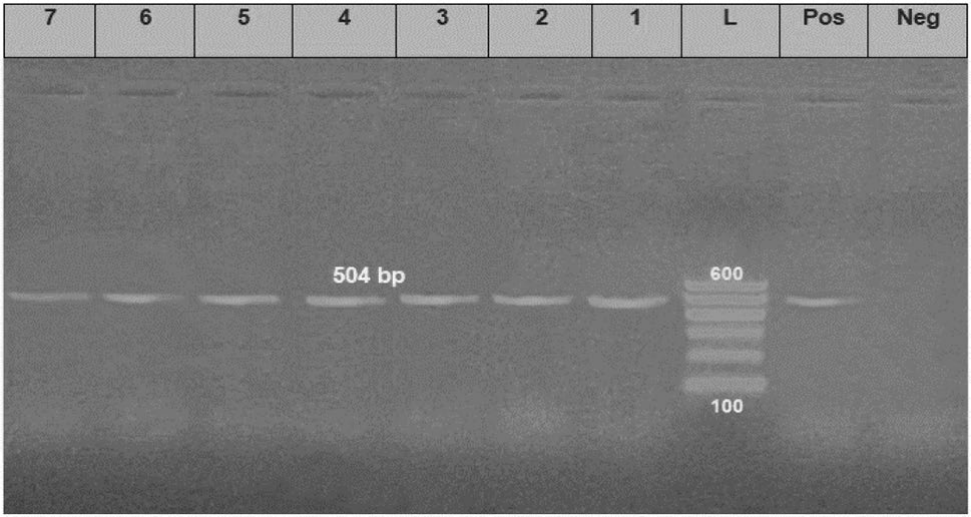
Electrophoretic pattern of primers targeting *opr*L gene. Lane L: 100 bp molecular weight ladder (Fermentas, Thermo Scientific, Germany); lane Pos: the positive control (Reference strain from Animal Health Research Institute in Dokki, Cairo, Egypt), and lane Neg: the negative control (template without DNA). Lanes 1–7: the specific DNA product (504 bp) amplified from representative isolates of *P. aeruginosa*.

**Figure 4:**
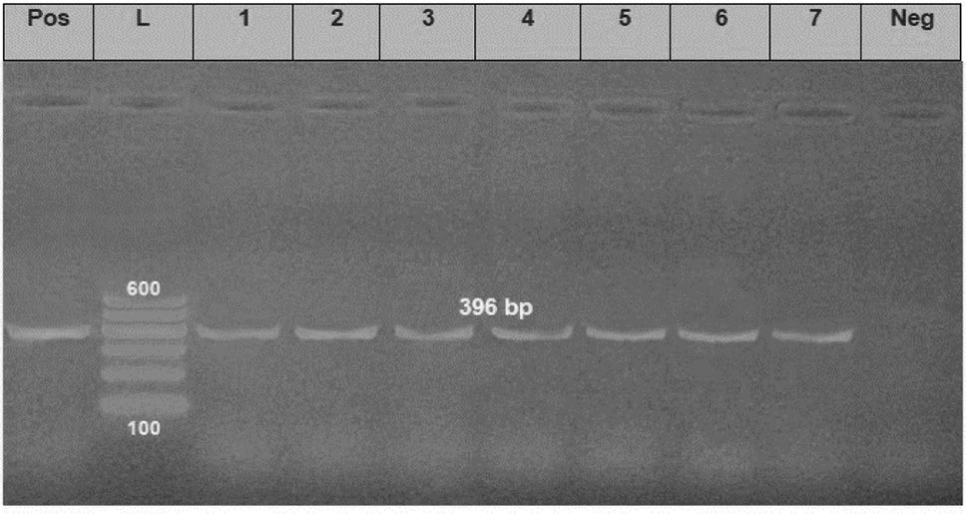
Electrophoretic pattern of primers targeting *tox*A gene. Lane L: 100 bp molecular weight ladder (Fermentas, Thermo Scientific, Germany); lane Pos: the positive control (Reference strain from Animal Health Research Institute in Dokki, Cairo, Egypt), and lane Neg: the negative control (template without DNA). Lanes 1–7: the specific DNA product (396 bp) amplified from representative isolates of *P. aeruginosa*.

**Figure 5:**
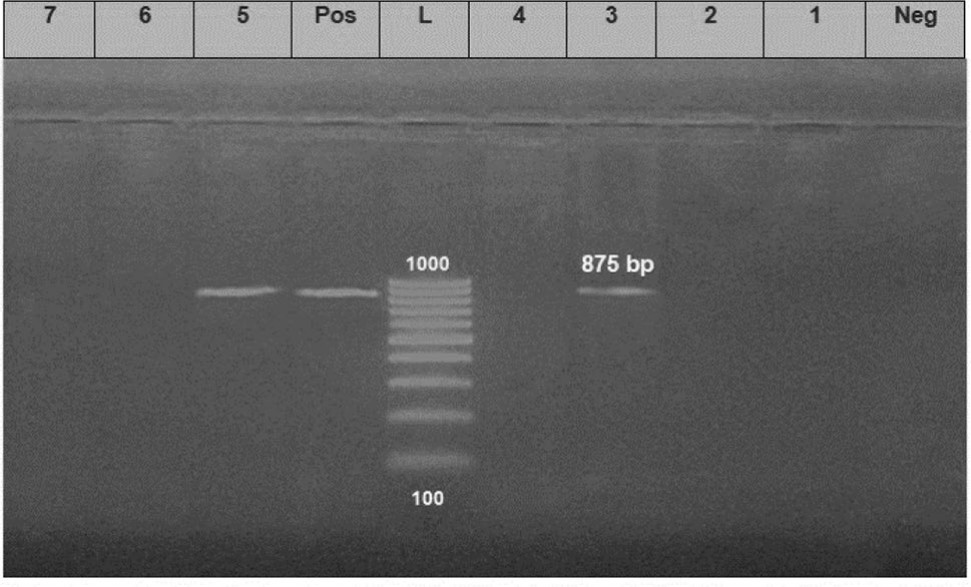
Electrophoretic pattern of primers targeting *phz*M gene. Lane L: 100 bp molecular weight ladder (Fermentas, Thermo Scientific, Germany); lane Pos: the positive control (Reference strain from Animal Health Research Institute in Dokki, Cairo, Egypt), and lane Neg: the negative control (template without DNA). Lanes 1–7: the specific DNA product (875 bp) amplified from representative strains of *P. aeruginosa*.

**Figure 6:**
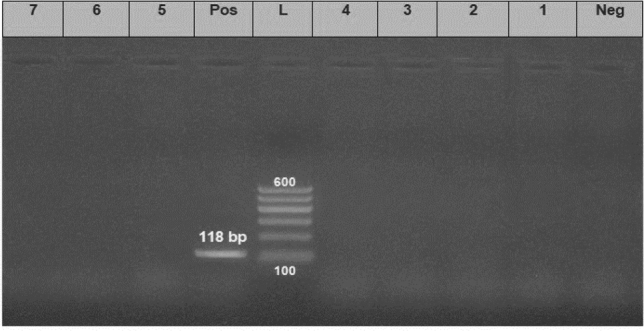
Electrophoretic pattern of primers targeting *exo*S gene. Lane L: 100 bp molecular weight ladder (Fermentas, Thermo Scientific, Germany); lane Pos: the positive control (Reference strain from Animal Health Research Institute in Dokki, Cairo, Egypt), and lane Neg: the negative control (template without DNA). Lanes 1–7: the specific DNA product (118 bp) amplified from representative strains of *P. aeruginosa*.

Several antibiotic-resistance and virulence genes associated with a natural outbreak of *P.* *aeruginosa* were selected based on the previous publications and were examined in ninety representative *P.* *aeruginosa* strains (the same strains that were tested for the antimicrobial susceptibility). The results showed that all tested strains harbored *opr*L gene (100%) with specific amplicons size of 504 bp (Fig. 3, Table 5), as well as *tox*A gene (100%) with amplicons size of 396 bp (Fig. 4, Table 5), while 20 strains (22.2%) were positive to the *phz*M gene with fragments size of 875 bp (Fig. 5, Table 5). On the contrary, none of the tested *P.* *aeruginosa* strains were positive for *exo*S gene (Fig. 6, Table 5). The statistical analysis revealed a significant difference in the prevalence of various virulence genes among the tested strains (*P* < 0.0001).

**Table 5:** The prevalence of virulence and antibiotic resistance genes among isolated *P. aeruginosa* strains from naturally infected *O. niloticus* and *C. gariepinus*.

Gene function	Gene acronym	Prevalence		Statistical analysis
		No	%	
Virulence genes	*oprL*	90	100	Chi-square value = 297.0 *P* value < .0001
	*toxA*	90	100	
	*exoS*	0	0	
	*phzM*	20	22.2	
Antibiotic-resistance Genes	*bla*_TEM_	75	83.3	Chi-square value = 2.4415 *P*-value 0.2950
	*tetA*	68	75.6	
	*bla*_CTX-M_	70	77.7

**Figure 7:**
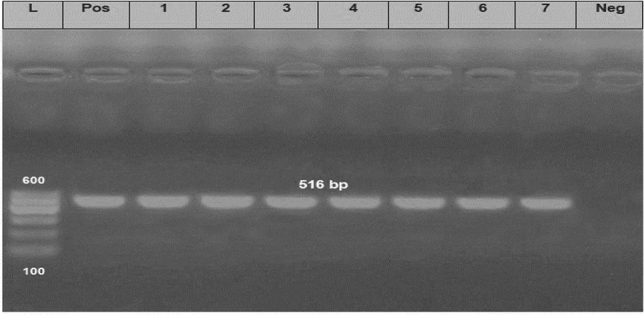
Electrophoretic pattern of primers targeting *bla*_*TEM*_ gene. Lane L: 100 bp molecular weight ladder (Fermentas, Thermo Scientific, Germany); lane Pos: the positive control (Reference strain from Animal Health Research Institute in Dokki, Cairo, Egypt), and lane Neg: the negative control (template without DNA). Lanes 1–7: the specific DNA product (516 bp) amplified from representative isolates of *P. aeruginosa*.

**Figure 8:**
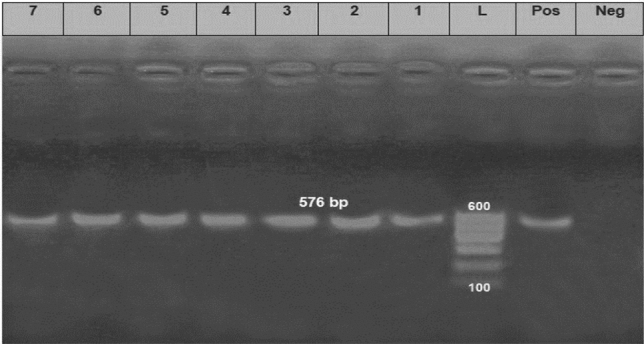
Electrophoretic pattern of primers targeting *tet*A gene. Lane L: 100 bp molecular weight ladder (Fermentas, Thermo Scientific, Germany); lane Pos: the positive control (Reference strain from Animal Health Research Institute in Dokki, Cairo, Egypt), and lane Neg: the negative control (template without DNA). Lanes 1–7: the specific DNA product (576 bp) amplified from representative strains of *P. aeruginosa*.

**Figure 9:**
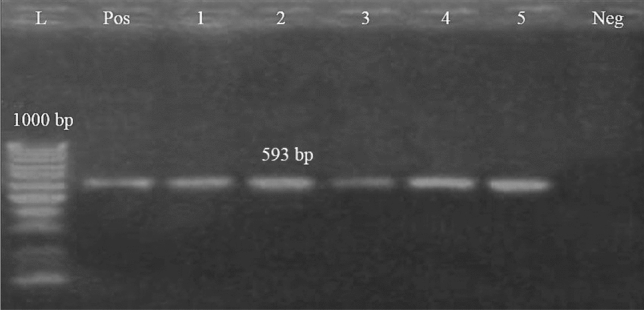
Electrophoretic pattern of primers targeting *bla*_*CTM*_ gene. Lane L: 100 bp molecular weight ladder (Fermentas, Thermo Scientific, Germany); lane Pos: the positive control (Reference strain from Animal Health Research Institute in Dokki, Cairo, Egypt), and lane Neg: the negative control (template without DNA). Lanes 1–5: the specific DNA product (593 bp) amplified from representative strains of *P. aeruginosa*.

Concerning the distribution of the antibiotic resistance genes, 75 strains (83.3%) were positive for the *bla*_*TEM*_ gene with a specific amplicon size of 516 bp, while 68 strains (75.6%) harboring *tet*A gene with a specific amplicon size of 576 bp, in addition, 70 strains (77.7%) harboring the *bla*_*CTX-M*_ gene and gave specific amplicon size of 593 bp (Table 5, Figs. 7,8 and 9).

In the present study, as illustrated in Tables 4 and 6; the isolated strains exhibited a remarkable resistance to amoxicillin (83.3%), cefotaxime (77.7%) tetracycline (75.6%), and gentamicin (67.6%). Furthermore, Fifty strains (50/90, 55.5%) showed multi-drug resistance to four antimicrobial agents: amoxicillin, cefotaxime, tetracycline, and gentamicin and harbored *bla*_TEM_, *bla*_CTX-M_, and *tet*A antimicrobial resistance genes. Out of which, forty-one strains harbored two virulence genes (*opr*L and *tox*A), while nine strains harbored three virulence genes (*opr*L, *tox*A, and *phz*M). The statistical analysis revealed a non-significant difference in the prevalence of various antimicrobial-resistance genes among the tested strains (*P* > 0.05). The distribution of the multi-drug resistance patterns, antimicrobial resistance genes, and virulence genes among the tested *P.* *aeruginosa* strains (*n* = 90) illustrated in Table 6.

**Table 6:** The distribution of the multi-drug resistance patterns, antimicrobial resistance genes and virulence genes among the tested *P. aeruginosa* strains (*n* = *90*).

Number of isolates	Phenotypic resistance patterns	Antimicrobial resistance genes	Virulence genes
41	Amoxicillin, cefotaxime, tetracycline, gentamycin	*bla*_*T*EM_*, bla*_CTX-M_*,* *tet*A	*opr*L*, exo*S
9	Amoxicillin, cefotaxime, tetracycline, gentamycin	*bla*_TEM_*, bla*_CTX-M_*, tet*A	*opr*L*, exo*S*, phz*M
11	Amoxicillin, tobramycin, tetracycline, gentamycin	*bla*_TEM_*, tet*A	*opr*L*, exo*S*, phz*M
5	Amoxicillin, cefotaxime, tobramycin	*bla*_TEM_*, bla*_CTX-M_	*opr*L*, exo*S
5	Amoxicillin, cefotaxime, tetracycline	*bla*_TEM_*, bla*_CTX-M_*, tet*A	*opr*L*, exo*S
4	Amoxicillin, cefotaxime, tobramycin, ofloxacin, levofloxacin	*bla*_TEM_, *bla*_CTX-M_	*opr*L*, exo*S
4	Cefotaxime, ofloxacin, Norfloxacin	*bla*_CTX-M_	*opr*L*, exo*S
2	Cefotaxime, tetracycline	*bla*_CTX-M_*, tet*A	*opr*L*, exo*S
9	Levofloxacin	–	*0pr*L*, exo*S

### Pathogenicity (Challenge trial)

**Figure 10:**
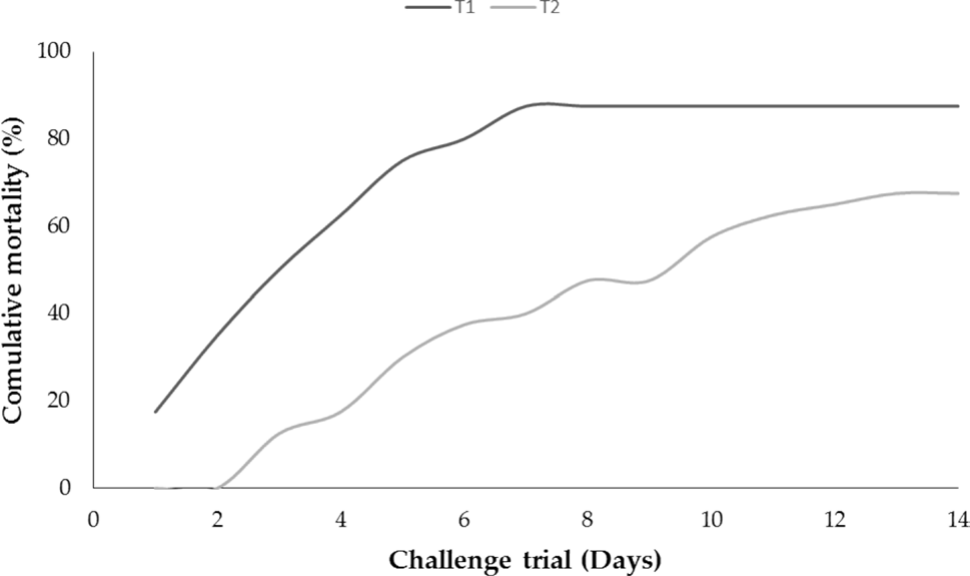
Cumulative mortality of *O. niloticus* subjected to intraperitoneal injection with 0.1 ml of virulent *P. aeruginosa* strain A (T1) and *P. aeruginosa* strain B (T2) at concentration of 3 × 10^7^ cells mL^-1^.

The clinical signs as well as fish morbidity and mortality were recorded in all experimental groups for 14 days post-challenge. The results demonstrated that the fish of the control group did not reveal any mortalities or pathological lesions, while those of the other groups displayed high mortalities and pathognomonic lesions of hemorrhagic septicemia, similar to those reported in naturally infected fish. It was noted that cumulative deaths were relatively associated with encoded virulence genes (Fig. 10), where the maximum mortality rate (87.5%) was recorded in T1 group, followed by T2 group (67.5%). Indeed, *O.* *niloticus* inoculated with a virulent strain A produced higher mortalities in a shorter time compared to those exposed to strain B. Approximately, 87.5% of infected fish in T1 group died within 7 days post-inoculation, while those of T2 group showed delayed mortality (67.5%) up to 12 days post-inoculation. Clinically, most of the experimentally infected fish showed exophthalmia, abdominal ascites, hemorrhagic gills, detached scales, and skin ulcers. The postmortem findings revealed that the challenged fish displayed typical signs of septicemia manifested by congested liver, enlarged spleen, and accumulation of serous bloody fluid in the abdominal cavity. In terms of bacteriology, *P.* *aeruginosa* was successfully isolated from skin ulcers and internal organs of dead and moribund fish, and the results confirmed that all isolates belong to *P.* *aeruginosa* based on biochemical characteristics and molecular typing.

## Discussion

*Pseudomonas* *aeruginosa* is a ubiquitous pathogen and is one of the principal causes of septicemia in freshwater fish, resulting in tremendous economic losses in fish producing sectors worldwide^41^. In the present study, a total of 285 fish samples (165 *O.* *niloticus* and 120 *C.* *gariepinus*) were collected randomly from private freshwater farms at Ismailia Governorate, Egypt, for clinical and bacteriological examinations. The results of clinical inspection revealed that 95 *O.* *niloticus* (95/165, 57.5%) and 65 *C.* *gariepinus* (65/120, 54.1%) were moribund and exhibited the typical signs of hemorrhagic septicemia**,** skin ulcerations, and fin rots. Moreover, internally the infected fish showed pale liver, engorged spleen, and abdominal dropsy with reddish ascetic exudates. These findings agreed with those obtained by Eissa et al.^1^ and Magdy et al.^42^ who reported that the postmortem findings due to *P.* *aeruginosa* infection were ascites, hepatic and renal necrosis, and congestion of all internal organs. The disease induced by *P.* *aeruginosa* is mainly associated with exophthalmia, skin discoloration, hemorrhage, detached scales, and abdominal distension^43^. Concerning the phenotypic characteristics of *P. aeruginosa*, all the isolated strains exhibited the typical phenotypic characteristics, culture characters, and biochemical characteristics of *P.* *aeruginosa.* These findings are nearly similar to those obtained by Aprameya^44^.

In the present study, *P.* *aeruginosa* isolated from the examined fish with a prevalence of 31.57%. The highest prevalence recorded in *O.* *niloticus* (32.72%) followed by *C.* *gariepinus* (30%), in agreement with Magdy et al.^42^ who recorded that the prevalence of *P.* *aeruginosa* in *O.* *niloticus* and *C.* *gariepinus* was 34.4% and 27.5%, respectively. Interestingly, none of the apparently healthy fish yielded *P.* *aeruginosa*, while the bacteria recovered from all moribund *O.* *niloticus* and *C.* *gariepinus* with a total prevalence of 56.8% and 55.4%, respectively. Regarding the distribution of *P.* *aeruginosa* in various internal organs, the liver was the most prominent infected organ, followed by the kidney and spleen, which is consistent with those reported by Eissa et al.^1^. Variations in prevalence could be related to the geographical distribution, environmental factors, host susceptibility, and the season of sample collection. Regarding the antimicrobial susceptibility testing, the examined strains were sensitive to colistin sulfate (100%), followed by norfloxacin (88.89%), while showed remarkable resistance to amoxicillin (83.3%), cefotaxime (77.7%) tetracycline (75.6.8%), and gentamicin (67.6%). Furthermore, Fifty strains (50/90, 55.5%) showed multi-drug resistance to four antimicrobial agents: amoxicillin, cefotaxime, tetracycline, and gentamicin and harbored *bla*_TEM_, *bla*_CTX-M_, and *tet*A antimicrobial resistance genes. Out of which, forty-one strains harbored two virulence genes (*opr*L and *tox*A), while nine strains harbored three virulence genes (*opr*L, *tox*A, and *phz*M). These findings consistent with those recorded by Eid et al.^45^ and Nasreen et al*.*^46^ who reported the resistance to tetracycline and gentamycin. Globally, several antimicrobial agents are frequently used for the treatment and/ or prevention of fish bacterial diseases. The indiscriminate use of antibiotics, as well as the emerging antibiotic resistance genes, could result in the occurrence of multi-drug resistant (MDR) strains^47–50^. Therefore, the routine application of antibiotic sensitivity testing is significant to select the specifically effective antibiotic and overcome such a problem^51–53^.

In the present study, the PCR results revealed that all the tested strains were positive for *opr*L and *tox*A genes, in agreement with Kenneth^54^. L- Lipoproteins refer to the outer membrane proteins associated with *P.* *aeruginosa* that enable the microorganism to resist the antiseptics and variable antimicrobial agents. L- lipoproteins are restricted to *Pseudomonads*, so it could be a reliable target used in both identification and virulence determination of *Pseudomonads* in clinical specimens^55^. Exotoxin A is an extracellular product of virulent *P.* *aeruginosa* that is encoded by the *tox*A gene in their chromosome. It acts by inhibition of protein-biosynthesis in the host cell, resembling the action of diphtheria toxin^56^.

On the contrary, none of the tested strains was positive for the *exo*S gene, while 20 strains were positive to the *phz*M gene. These results agreed with those recorded by Nowroozi et al.^57^. The presence of the *phz*M gene in 22.2% of the examined strains indicates their ability to produce a phenazine toxin, which in turn enhances their survival rate and colonization to the host even under adverse environmental circumstances^58,59^. Concerning the distribution of the antibiotic-resistance genes: the examined strains harbored *bla*_TEM_, *bla*_CTX-M_, and *tet*A genes with a total prevalence of 83.3%, 77.7%, and 75.6%, respectively, in agreement with Ndi and Barton^60^, and Ishida et al.^61^. The presence of these genes explains the phenotypic resistance of the tested *P.* *aeruginosa* strains to cefotaxime, amoxicillin, and tetracycline^62^.

Regarding the pathogenicity trial, fish challenged with *P.* *aeruginosa* provided high mortality rates in direct proportion to the encoded virulence genes and showed similar signs of septicemia found in the naturally infected one. Our results are in a good agreement with Magdy et al.^42^ who noticed marked histopathological alterations in *C.* *gariepinus* experimentally challenged with *P.* *aeruginosa*. The present findings paralleled with those reported by Devakumar et al.^63^ who observed degenerative changes in all tissues (brain, ovary, liver, and gills) of crabs infected with *P.* *aeruginosa*. Likewise, Derwa et al.^64^ demonstrated that *O.* *niloticus* experimentally infected with *P.* *aeruginosa* clinically suffered from exophthalmia, scale losses, skin ulcerations, and external hemorrhages over all the body surfaces and at the base of fins, while, internally they showed a distended gall bladder, congestion, and enlargement of liver, spleen, and kidneys. The degenerative changes could be related to the fatal and subversive influence of bacterial toxins, enzymes, and bioactive extracellular components, which promote tissue damage and cell necrosis^23^. The elaboration of exotoxins: groups of proteins, is the most crucial factor in *P.* *aeruginosa* pathogenicity. The exotoxins are proven to induce liver necrosis, hemorrhage, and renal nephrosis^65^.

In conclusion, *P.* *aeruginosa* is one of the major recurrent emerging pathogens frequently isolated from *O.* *niloticus* and *C.* *gariepinus*. The recovery of multi-drug resistant (MDR) strains of *P.* *aeruginosa* gave a warning to the potential and proper use of antibiotics. The most frequent antibiotic-resistance genes associated with *P.* *aeruginosa* isolated from fish are *bla*_TEM_, *tet*A, and *bla*_CTX-M_ genes that induced resistance patterns to amoxicillin, tetracycline, and cefotaxime, respectively. Routine application of the antimicrobial susceptibility testing is necessary to prevent the emergence of antibiotic-resistant strains of potential public health concern*.* *opr*L and *tox*A genes are the most predominant virulence genes associated with *P.* *aeruginosa*.
